# Intra- and extracellular β-amyloid overexpression via adeno-associated virus-mediated gene transfer impairs memory and synaptic plasticity in the hippocampus

**DOI:** 10.1038/s41598-019-52324-0

**Published:** 2019-11-04

**Authors:** Stefania Forner, Alessandra C. Martini, G. Aleph Prieto, Cindy T. Dang, Carlos J. Rodriguez-Ortiz, Jorge Mauricio Reyes-Ruiz, Laura Trujillo-Estrada, Celia da Cunha, Elizabeth J. Andrews, Jimmy Phan, Jordan Vu Ha, Allissa V. Z. D. Chang, Yona Levites, Pedro E. Cruz, Rahasson Ager, Rodrigo Medeiros, Masashi Kitazawa, Charles G. Glabe, Carl W. Cotman, Todd Golde, David Baglietto-Vargas, Frank M. LaFerla

**Affiliations:** 1 Institute for Memory Impairments and Neurological Disorders, University of California, Irvine, Irvine, CA 92697 USA; 2 Department of Neurobiology and Behavior, University of California, Irvine, Irvine, CA 92697 USA; 3 Department of Neurology, School of Medicine, University of California, Irvine, Irvine, CA 92697 USA; 4 Department of Neuroscience, University of Florida, Gainesville, FL 32610 USA; 5 Department of Molecular Biology and Biochemistry, University of California, Irvine, Irvine, CA 92697 USA; 6 Department of Medicine, University of California, Irvine, Irvine, CA 92697 USA; 7 Clem Jones Centre for Ageing Dementia Research, Queensland Brain Institute, The University of Queensland, Brisbane, QLD 4072 Australia

**Keywords:** Cognitive ageing, Neural ageing

## Abstract

Alzheimer’s disease (AD), the most common age-related neurodegenerative disorder, is currently conceptualized as a disease of synaptic failure. Synaptic impairments are robust within the AD brain and better correlate with dementia severity when compared with other pathological features of the disease. Nevertheless, the series of events that promote synaptic failure still remain under debate, as potential triggers such as β-amyloid (Aβ) can vary in size, configuration and cellular location, challenging data interpretation in causation studies. Here we present data obtained using adeno-associated viral (AAV) constructs that drive the expression of oligomeric Aβ either intra or extracellularly. We observed that expression of Aβ in both cellular compartments affect learning and memory, reduce the number of synapses and the expression of synaptic-related proteins, and disrupt chemical long-term potentiation (cLTP). Together, these findings indicate that during the progression AD the early accumulation of Aβ inside neurons is sufficient to promote morphological and functional cellular toxicity, a phenomenon that can be exacerbated by the buildup of Aβ in the brain parenchyma. Moreover, our AAV constructs represent a valuable tool in the investigation of the pathological properties of Aβ oligomers both *in vivo* and *in vitro*.

## Introduction

Alzheimer’s disease (AD) is the most common age-related neurodegenerative disorder, afflicting approximately 5.5 million individuals in the USA and with a new case developing every 66 seconds^1^. The pathophysiologic mechanisms of AD begin long before symptom onset provides an opportunity to apply advanced imaging and biomarker methods to diagnose individuals during the preclinical stages of neurodegeneration. All AD brains are characterized by two main hallmarks: plaques, consisting of extracellular deposits of β-amyloid (Aβ), and tangles, aggregates of hyperphosphorylated tau^2^. A fundamental mechanism in AD pathogenesis is synapse failure^3,4^, and several studies suggest that Aβ oligomers can interact with different proteins, ultimately leading to synapse toxicity, alterations in long-term potentiation (LTP), and cognitive impairments^5–8^. Also, significant synaptic loss is already observed in patients with mild cognitive impairment (MCI), and progressive loss is the most robust hallmark that correlates with cognitive decline in AD^9,10^. However, the underlying molecular mechanisms that impair the synaptic function, and cause their loss at early stages of the disease, are poorly understood. Dendritic spines are specialized anatomical structures in neuronal cells that serve as the postsynaptic component for the vast majority of CNS synapses, and are major sites of processing and storage of information in the brain^2^. Structural changes at dendritic spines underlie learning and memory processes in the brain, and alterations in spine structure and function might lead to cognitive impairments^4^.

A continuing debate is whether intra- or extracellular Aβ is more deleterious to the brain. It is known that intraneuronal Aβ accumulation occurs early in adult life in basal forebrain cholinergic neurons, increasing with both aging and AD in the presence of intermediate and large oligomeric states^11^. The early intraneuronal accumulation has also been observed in transgenic rats McGill-R-Thy1-APP, which harbor the human APP751 transgene with the Swedish and Indiana mutations under the control of the murine Thy1.2 promoter^12^. In these animals, the intraneuronal pathology was due to a mixture of APP, CTFs and a considerable amount of Aβ, leading to deleterious effects in the CNS before amyloid plaques develop. Alternatively, additional studies suggest that intraneuronal Aβ precedes plaques in brain areas affected early in the disease^13^, is associated with synaptic deficits^14^, and can be secreted in a prion-like manner^15^. There is evidence of the accumulation of extracellular Aβ in an age-dependent manner^16^. Exogenous oligomers accumulate particularly at synaptic spines, and presynaptic sites can also be targeted^17^. However, as plaques correlate poorly with AD-related dementia and little is known about the effects of intracellular Aβ on cognitive function, we asked whether these two distinct accumulation sites could have different effects on the development of pathology.

Several animal models of AD employ an approach based on the overexpression of mutant human amyloid precursor protein (APP) or presenilin to increase Aβ expression^18^. Although these have been very useful and are still fundamental to the understanding of AD development, increasing the expression of Aβ42 via AAV-mediated gene transfer has been demonstrated useful in developing AD animal models^19,20^. In this study, we used this method to overexpress Aβ42 oligomers in mouse hippocampus and neuronal cultures and to analyze their effect in synaptic function and cognitive impairments. Overall, our findings reveal a faster yet physiologically relevant model that show that synaptic impairment and cognitive decline initiates as soon as Aβ accumulates in the intracellular compartment in AD.

## Results

### Characterization of Aβ expression following incubation and injection of AAV-BRI-Aβ42 and AAV-UBI-Aβ42 vectors

The detailed description of generating AAV constructs used here was reported previously^19–21^. AAV vectors encoding BRI-Aβ cDNAs, fusions between human Aβ peptides and the BRI protein (known to be associated with amyloid deposition in British familial dementia), are able to promote high-level expression of Aβ peptide in the absence of APP overexpression^19^. AAV-BRI-Aβ42 and AAV-UBI-Aβ42 were created to facilitate the expression of extracellular and intracellular Aβ, respectively^22^.

**Figure 1:**
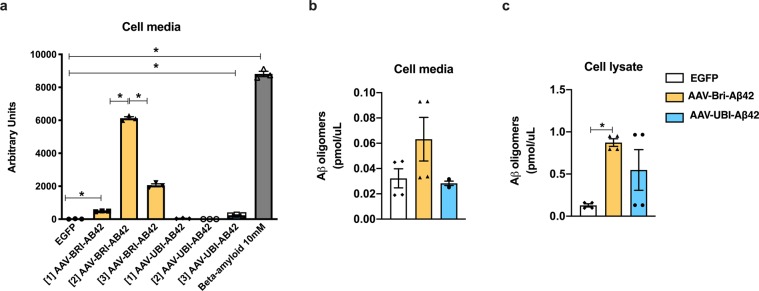
Expression of Aβ by AAV-BRI-Aβ42 and AAV-UBI-Aβ42 in hippocampal neuronal cell cultures. (**a**) Total Aβ42 levels in the media after AAV constructs incubation. AAV-BRI-Aβ42: [1] 4.7 × 10^10^ genome particles/ml, [2] 9.3 × 10^10^ genome particles/ml, and [3] 2.8 × 10^11^ genome particles/ml. AAV-UBI-Aβ42: [1] 1.5 × 10^10^ genome particles/ml, [2] 7.7 × 10^10^ genome particles/ml, [3] 4.6 × 10^11^ genome particles/ml. AAV-BRI-Aβ42 [1] and [2] promoted a 37-fold and 465-fold increase and AAV-UBI-Aβ42 [3] promoted an 18-fold increase in Aβ42 levels when compared to those elicited by EGFP. In comparison to AAV-BRI-Aβ42 [2], the concentration [3] promoted a 3-fold decrease in the levels of Aβ42. Compared to EGFP, Aβ 10 mM elicited a 669-fold increase (*p < 0.0001). (**b**) No significant change was observed in Aβ42 oligomers in the cell media after incubation of either AAV constructs. (**c**) AAV-BRI-Aβ42 promoted a significant increase in the levels of Aβ42 oligomers in the cell lysate compared to EGFP (*p < 0.0001).

Prior to *in vivo* testing, we incubated hippocampal neuronal cell cultures with AAV constructs encoding BRI-Aβ42 or UBI-Aβ42 fusion proteins in order to determine the optimal concentration and efficacy for each construct. Exogenous synthetic oligomers of Aβ were used as a positive control. We detected significant levels of Aβ42 in the culture medium using three concentrations of BRI-Aβ42, in contrast to the UBI-Aβ42 and EGFP control AAV constructs (Fig. 1a). We also measured the expression of Aβ oligomers in cell media and lysate of cell cultures. There is no significant increase in the cell media after incubation of either AAV constructs, even though there is an increased trend in the AAV-BRI-Aβ42 in accordance with what has been observed in Fig. 1a. We observed an increase in the levels of oligomers in the cell lysate after incubation with AAV-BRI-Aβ42 compared to EGFP (Fig. 1c). With these results we confirmed the ability of these constructs to promote the overexpression of Aβ peptides.

**Figure 2:**
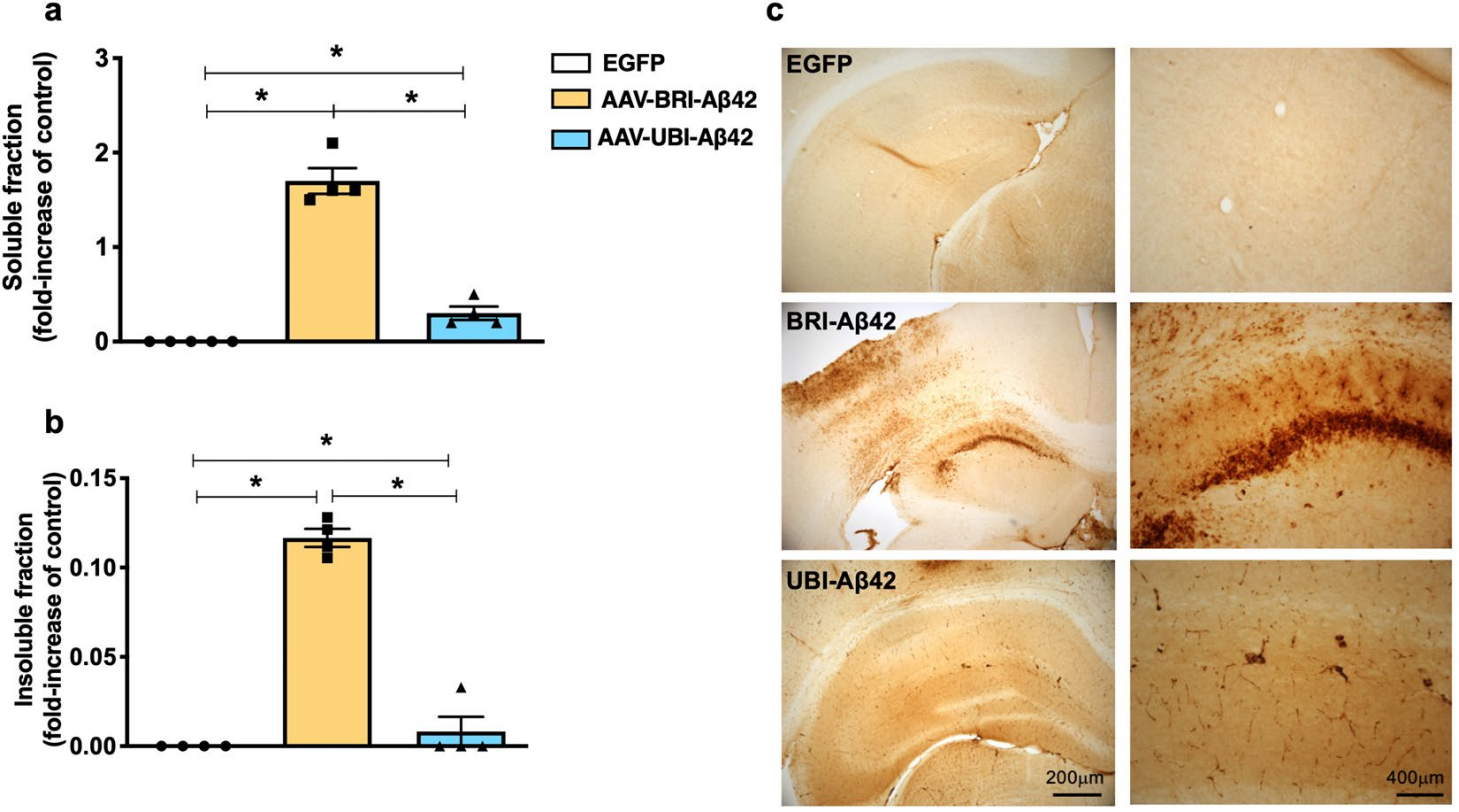
Expression of Aβ by AAV-BRI-Aβ42 and AAV-UBI-Aβ42 in mice hippocampus. (**a**,**b**) Both soluble and insoluble fractions of the mouse hippocampus show an increase in Aβ42 levels after AAV-BRI-Aβ42 or AAV-UBI-Aβ42 transfection (*p < 0.0001). (**c**) Light microscopy images of the hippocampus (CA1 region) immunostained with anti-Aβ antibody (6E10) of ntg mice treated with EGFP, AAV-BRI-Aβ42 or AAV-UBI-Aβ42.

Next, we tested the AAV-BRI-Aβ42 and AAV-UBI-Aβ42 expression *in vivo* by determining the Aβ42 relative levels in the soluble and insoluble fractions of mice hippocampus after AAVs injection. Mice were divided into 3 cohorts: AAV-BRI-Aβ42-treated, AAV-UBI-Aβ42-treated or AAV-EGFP-treated. Each subject received bilateral intrahippocampal injection of a single specific AAV construct, and the brains were collected and analyzed 3 months later. The overexpression of BRI-Aβ42 construct resulted in higher expression of both soluble and insoluble Aβ42 as compared to the UBI-Aβ42 construct, while there was no detectable Aβ following EGFP incubation (Fig. 2a,b). Immunostaining for 6E10 in the BRI-Aβ42 construct demonstrated a high amyloid deposition in the hippocampus. However, animals that received the UBI-Aβ42 presented distinct neuronal processes staining with mild intraneuronal accumulation of Aβ (Fig. 2c), without accumulation of Aβ deposits^23^.

### Hippocampal Aβ expression promoted by AAV constructs leads to impaired cognition

**Figure 3:**
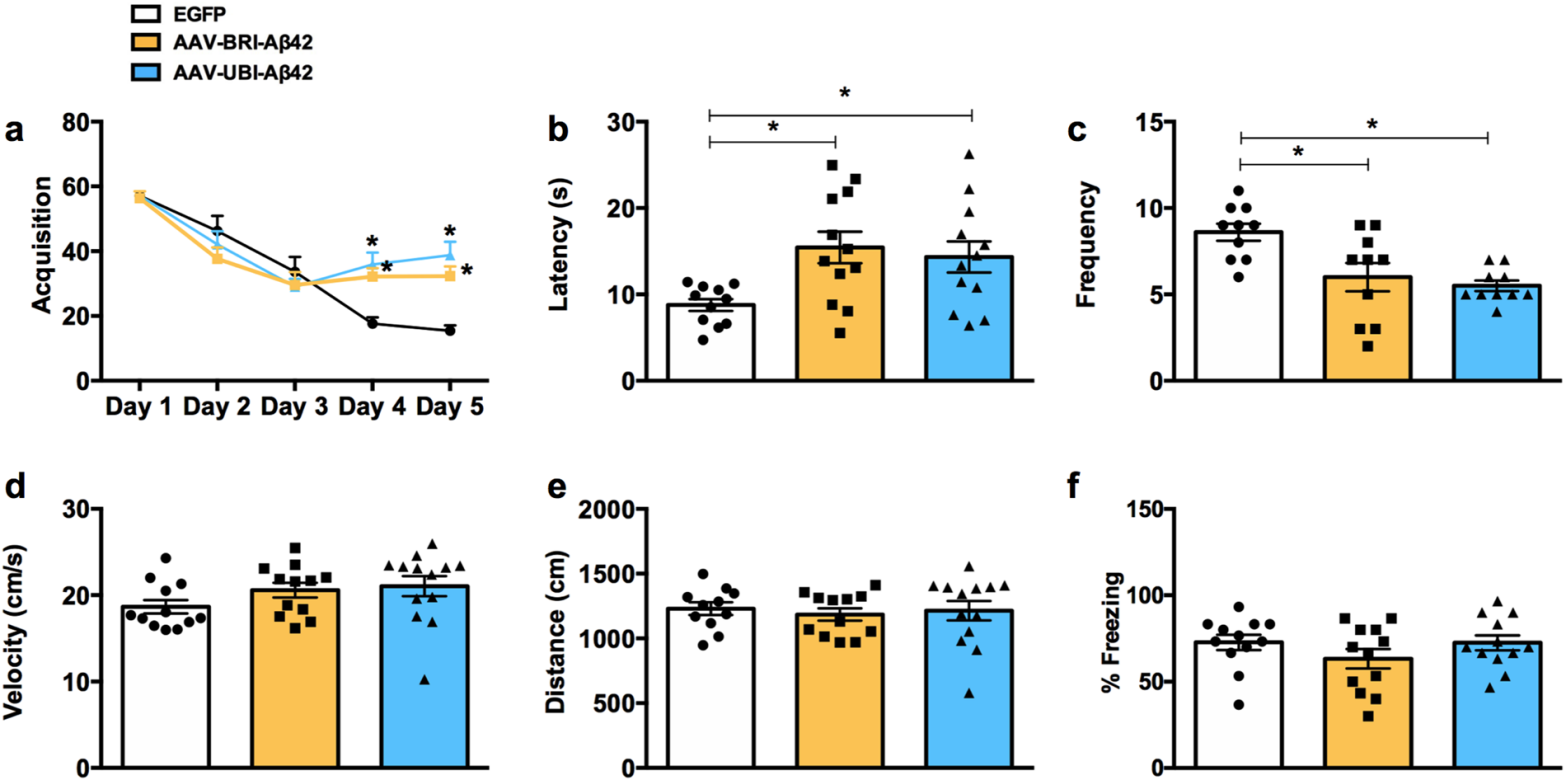
Aβ AAV-mediated gene transfer impair cognition and LTP in ntg mice. (**a**) Mice were trained on the spatial reference version of the Morris water maze at 8–9 months of age. Acquisition curves for the 5 days of training show significant differences in groups treated with AAV-BRI-Aβ42 and AAV-UBI-Aβ42 compared to EGFP (*p < 0.0001). (**b**) Animals in the AAV-BRI-Aβ42 and AAV-UBI-Aβ42 groups had an increase in the latency to reach the hidden platform compared to EGFP group (175.82% ± 20.86 and 156.00% ± 20.13, respectively; *p = 0.0127). (**c**) A significant decrease in frequency was observed in the AAV-BRI-Aβ42 and AAV-UBI-Aβ42 groups (30.22% ± 9.49 and 36.06% ± 3.58, respectively; *p = 0.0016). (**d**) No differences were observed between groups in distance or velocity behavior. Values represent the mean ± S.E.M (n = 10 per group).

Animals treated with both Aβ AAV constructs presented significant cognitive impairment, measured by performance in the Morris Water maze test (Fig. 3). In this evaluation, both groups took more time to find the hidden platform – as demonstrated by latency (Fig. 3b) – and also crossed the platform fewer times (Fig. 3c). The injection of both vectors did not affect motor skills, as demonstrated by velocity and distance evaluation (Fig. 3d,e). These results are significant and demonstrate the importance of both intra- and extracellular Aβ in the development of spatial cognitive impairments. There are no significant changes with contextual fear conditioning (Fig. 3f).

### *In vitro* synaptic function is altered by both BRI-Aβ42 and UBI-Aβ42

**Figure 4:**
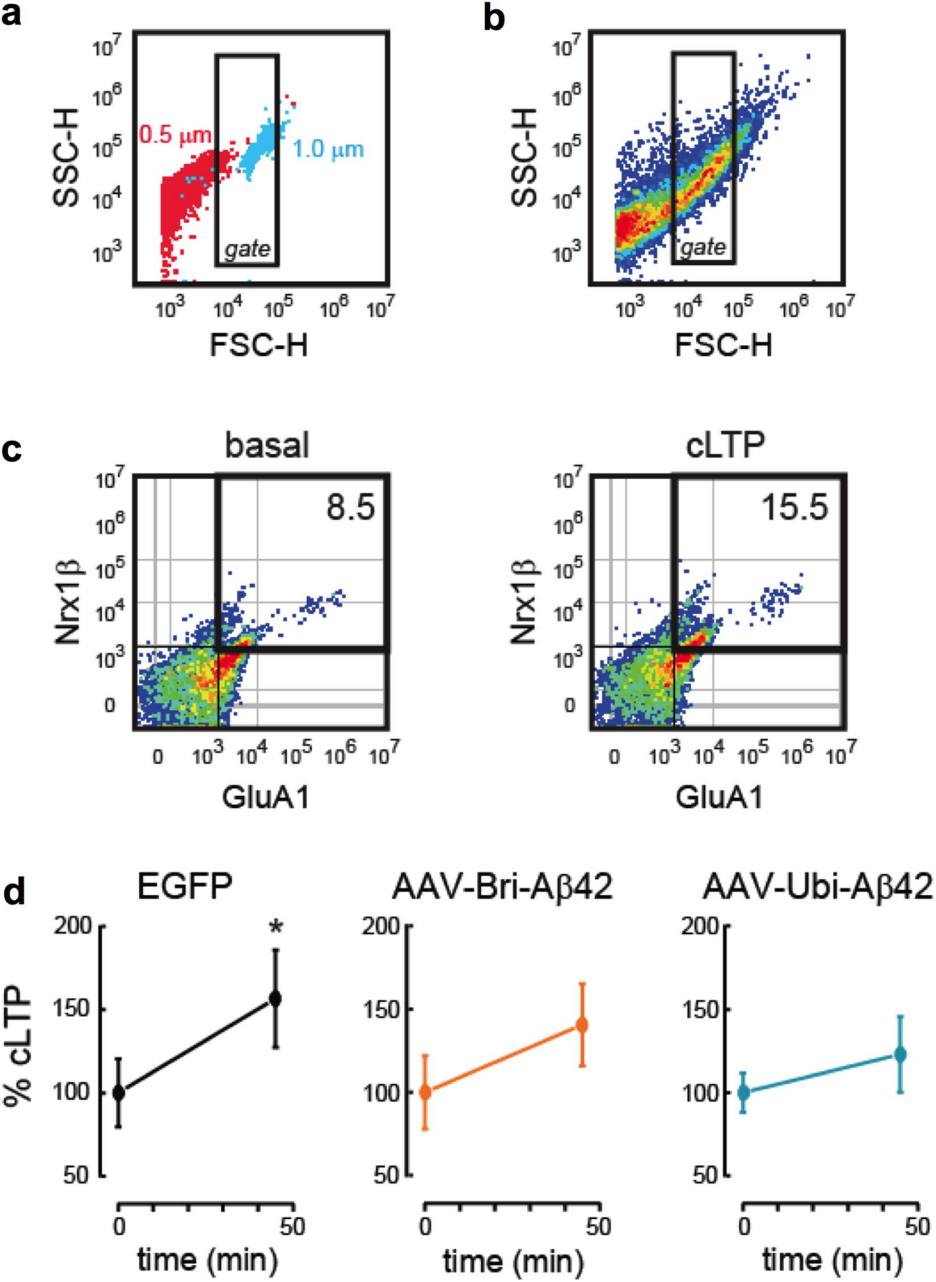
AAV-BRI-Aβ42 and AAV-UBI-Aβ42 induce functional, morphological, and structural synaptic alterations. (**a**) Flow cytometry FASS-LTP identifies synaptosomes by size. Based on calibrated beads, we set a threshold in the forward scatter (FSC-H) channel, as well as a gate region. We excluded small particles having a size equivalent to 0.5 µm calibrated beads (red particles), while selecting particles of ~ 1.0 µm calibrated beads (blue particles). (**b**) Forward-Side (FSC-SSC) profile of particles in the synaptosomal P2 fraction isolated from the hippocampus. The inside rectangle (gate) selects putative synaptosomes according with to size (~1.0 µm = size-gated synaptosomes). (**c**) In size-gated synaptosomes, FASS-LTP identifies potentiated synapses by tracking GluA1 and Nrx1β surface staining. To induce cLTP in samples from each experimental group (EGFP, AAV-BRI-Aβ42 and AAV-UBI-Aβ42 samples were run in parallel), synaptosomal P2 fractions maintained in Mg2+ -free external solution were sequentially stimulated using 500 µM glycine (15 min) and 37 mM KCl (30 min). As controls, equivalent volumes of external solution were added to a parallel set of synaptosomal fractions maintained in external solution (basal). Representative two-color parameter plots show GluA1 (x-axis) and Nrx1β (y-axis) surface levels in basal and cLTP conditions. Thresholds for endogenous/non-specific fluorescence for each marker were set by staining with secondary antibodies only. (**d**) Values normalized to the basal state in each experimental group, mean ± SEM. Basal vs cLTP: EGFP, *p = 0.041 (n = 6); AAV- BRI-Aβ42, P = 0.244 (n = 7); AAV-UBI-Aβ42, P = 0.461 (n = 6).

As Aβ is known to cause dendritic spine density and synaptic function changes^24^, we next examined if the cognitive impairments observed in our animals were associated with functional or structural changes in dendritic spines or specific synaptic markers. To determine possible mechanisms by which the AAV-driven expression of Aβ42 could impair cognition, we evaluated the effect of BRI-Aβ42 and UBI-Aβ42 expression on long-term potentiation (LTP), the best-known cellular correlate of memory^25^. We used FASS-LTP, a flow cytometry-based method that quantifies surface GluA1 expression in isolated synaptosomes following chemical LTP (cLTP)^26–28^. Synaptosomal cLTP is based on application of the NMDA receptor co-agonist glycine, which facilitates NMDA receptor activation^29^. By flow-cytometry, FASS-LTP selectively and reliably identifies synaptosomal particles (subset of particles of ~1.0 μm) using calibrated beads (Fig. 4a,b), and quantifies potentiated synaptosomes by extracellular labeling of the AMPA receptor subunit GluA1 and neurexin-1β (Nrx1β)^26,27^, a presynaptic adhesion molecule stabilized at the membrane surface by synaptic activity^30^. GluA1 and Nrx1β double-labeling ensures the analysis of intact synaptosomes that contain both pre- and postsynaptic elements. In synaptosomes of mice treated with the control AAV-EGFP vector, cLTP stimulation increased the proportion of synaptosomes expressing both GluA1 and Nrx1β at the surface (GluA1 + Nrx1β+), relative to non-stimulated synaptosomes (basal condition, Fig. 4d). In contrast, the levels of potentiated GluA1 + Nrx1β+ synaptosomes were similar in the basal and cLTP conditions in the BRI-Aβ42 and UBI-Aβ42 groups (*P* > 0.05, Fig. 3d), thereby indicating that both BRI-Aβ42 and UBI-Aβ42 impair LTP mechanisms directly at the synapse.

### Both BRI-Aβ42 and UBI-Aβ42 drive synapse loss *in vivo*

**Figure 5:**
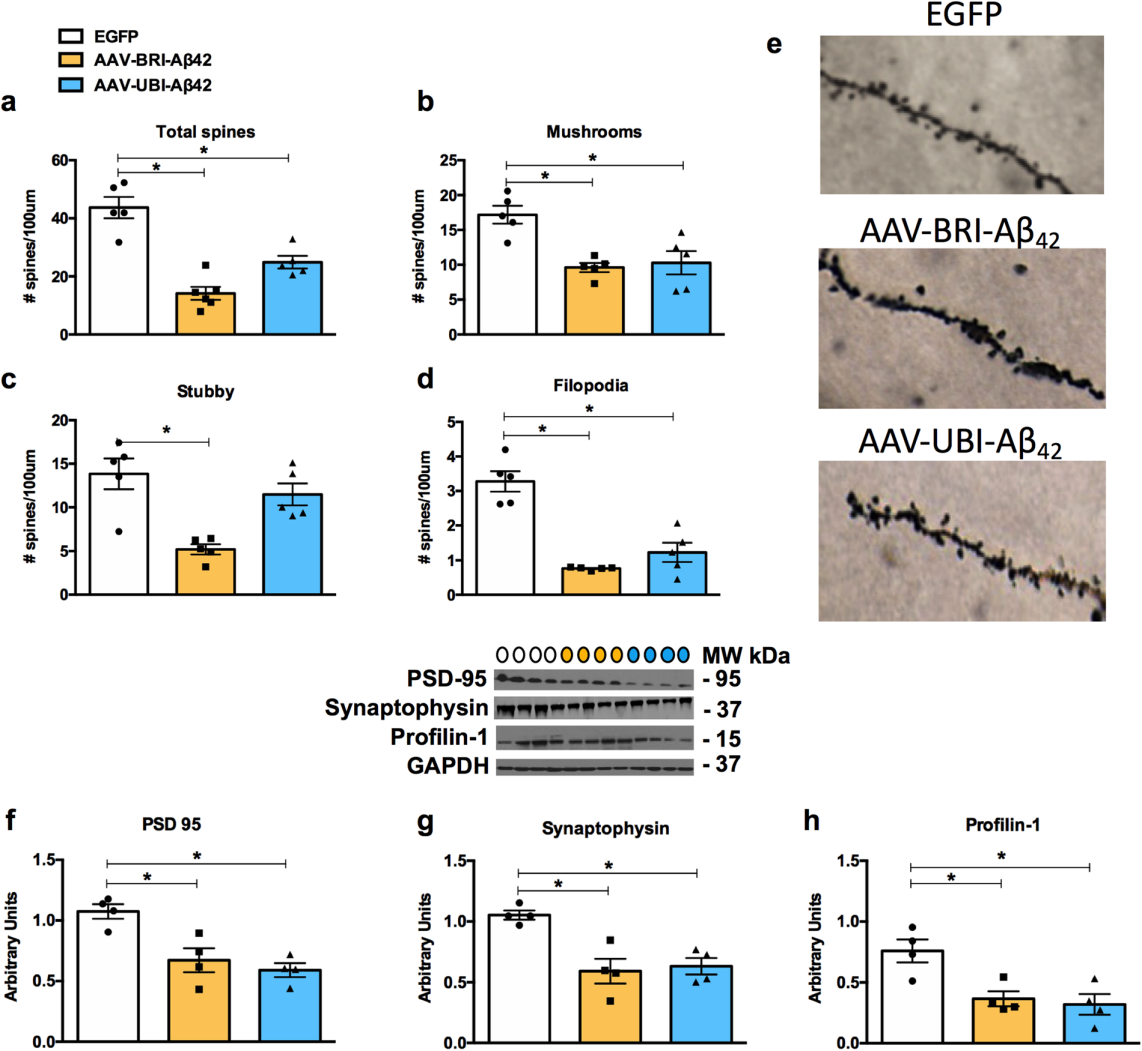
Impairments in synaptic number and synaptic proteins are related to Aβ overexpression via AAV-mediated gene transfer. (**a**–**d**) Stereological quantification showed a significant decrease in total spines for the AAV-BRI- Aβ42 and AAV-UBI- Aβ42 groups (67.65% ± 5.06; 43.02% ± 4.93; *p < 0.0001) when compared to AAV-EGFP-transfected mice. There was also a significant decrease in mushroom (BRI-Aβ42: 44.09% ± 4.83; UBI-Aβ42: 40.13% ± 9.78; *p = 0.0021), stubby (BRI-Aβ42: 62.34% ± 4.20, *p = 0.0015) and filopodia-like spines (BRI-Aβ42: 76.72% ± 0.59; UBI-Aβ42: 62.53% ± 8.42; *p < 0.0001) compared to the number observed in EGFP-transfected mice. Notably, there was no significant difference in stubby spines in the AAV-UBI-Aβ42 group. The values represent the mean ± SEM (n = 5 per group). (**e**) Light microscopic 3D reconstruction images of dendritic spines in the CA1 subfield in AAV-EGFP, AAV-BRI- Aβ42 and AAV-UBI- Aβ42 mice. (**f**–**i**) Immunoblot analysis of hippocampal homogenates of 8–9 month-old mice, normalized to GAPDH and expressed as arbitrary units, showing a significant reduction in the expression of PSD-95 (BRI-Aβ42: 37.20% ± 9.19; UBI-Aβ42: 44.80% ± 5.35; *p = 0.0028), synaptophysin (BRI-Aβ42: 43.69% ± 9.75; UBI-Aβ42: 39.82% ± 6.46; *p = 0.0030) and profilin-1 (BRI-Aβ42: 51.87% ± 8.00; UBI-Aβ42: 42.04% ± 11.13; *p = 0.0075) compared to the EGFP group. Quantification of western blots was performed via densitometric analysis and is presented as arbitrary units, normalized to GAPDH. Colored circles represent the groups tested: white - EGFP; yellow - BRI-Aβ42; blue - UBI-Aβ42. Values represent the mean ± SEM (n = 4 per group).

Consistent with the suppression of synaptosomal cLTP by BRI-Aβ42 and UBI-Aβ42, we found that our AAV constructs reduced the total number of spines (Fig. 5a), as well as that of stable mushroom spines (Fig. 5b). The BRI-Aβ42 construct also significantly reduced stubby (Fig. 5c) and filopodia-like spines (Fig. 5d), whereas the UBI-Aβ42 construct did not affect the stubby spines, i.e. there was a higher number of these immature spines in this group. In addition, we observed reduced protein levels of the pre- and postsynaptic markers PSD-95 and synaptophysin, respectively (Fig. 5f,g), as well as of profilin-1 (Fig. 5h), an actin cytoskeleton protein, suggesting that impairments in synaptic function and strength could be related to intra- and extracellular Aβ.

## Discussion

The findings of this study imply that intra- and extracellular Aβ accumulation mediated by AAV-gene transfer can promote deleterious effects on synaptic and cognitive functions. Our study indicates that these AAV constructs of Aβ42 induce memory impairments, alongside reductions in the number of spines and of proteins related to synaptic function and LTP. These results are significant as they demonstrate that mild intraneuronal accumulation of Aβ42 (UBI-42) is as potent as combined intraneuronal and extracellular accumulation (BRI-42), significantly impacting our understanding of the detrimental aspects of AD.

The view that insoluble Aβ fibrils are the major factor in AD pathogenesis was firmly held until prefibrillar soluble Aβ oligomers were shown to be more detrimental in some experimental settings^31–35^. These oligomers can interact with glutamate receptors, dysregulating calcium influx and also altering spine morphology and density^36^. Moreover, when extracted directly from AD brains and injected into rodent brains, they can inhibit LTP, enhance long-term depression (LTD), and reduce dendritic spine numbers^37^. Although the potential role of extracellular Aβ oligomers is more well-established, there is also evidence that intracellular Aβ and its deposition precede the formation of plaques in animal models and human brains^38^. We observed a significant increase in both soluble and insoluble fractions of Aβ, particularly with the BRI-Aβ42 construct. Moreover, this construct also induced the formation of plaques as well as intraneuronal accumulation of Aβ in the CA1 area of the hippocampus.

The first reports describing the existence of intracellular Aβ appeared shortly after the original identification of Aβ as the main component of plaques. Since then, other studies have provided evidence for intracellular Aβ accumulation in transgenic mouse brain and in *post-mortem* brain samples from AD and Down syndrome patients. A rat model with a doubly mutated APP, driven by a Thy1.2 promoter highly expressed in neurons, triggered the accumulation of intraneuronal human Aβ in 2–3-month-old rats, coinciding with cognitive impairments and pre-plaque generation^39^. Further evidence linking intraneuronal Aβ accumulation to cognitive deficits and synaptic dysfunction came from transgenic mouse models^38,40–42^. However, the overexpression of specific genes and the subsequent formation of plaques and/or tau pathology in such models make it hard to attribute the associated cognitive impairments they promote exclusively to the presence of intracellular Aβ. Our results are significant because, through the promotion of both intra- and extracellular Aβ, we can conclude that the accumulation of Aβ peptides, even in young mice, is sufficient to promote behavioral and synaptic impairments.

Synaptic deficits and loss are the pathological hallmarks that best correlate with the progressive cognitive decline observed in AD patients^43–45^. Here, we have demonstrated that synaptic function as measured by FASS-LTP is affected very early. We used FASS-LTP, an approach to evaluate chemically-induced LTP directly in isolated synaptosomes. FASS-LTP identifies the subset of synaptosomes that are double-labeled for surface GluA1 and Nrx1β (GluA1 + Nrx1β +). Importantly, the increase in surface GluA1 + Nrx1β+ levels after cLTP stimulation is sustained, and mechanistically parallels the facilitation of synaptic transmission following electrically induced LTP (*e*.*g*., dependence on NMDAR and CaMKII), as previously shown^26,27^. Specifically, the generation of cLTP was reduced significantly in the presence of either intra- and extracellular Aβ. Our results are in accordance with recent findings, whereas LTP was blocked by the intracellular injection of Aβ42 into hippocampal pyramidal cells^46^, and by extracellular Aβ at the CA3-CA1 synapses of APP-knockout mice^47^. Moreover, the Aβ42-induced impairment of glutamatergic synaptic function is dependent on its internalization and intracellular accumulation^48^.

Information storage underlying learning and memory, as well as signal transduction at excitatory synapses, develop at the postsynaptic density (PSD)^49,50^. Within PSDs, the most abundant scaffolding protein is PSD-95, which is known to play key roles in synaptic plasticity^51,52^. In the pre-synapse, the loss of synaptophysin is one of the best brain correlates of cognitive decline in AD^53^, occurring early in the development of disease and accompanied by increased APP and hyperphosphaorylated tau expression in neurons of the hippocampus and entorhinal cortex^54^. Interestingly, the reduction in synaptic density is more pronounced within immature and mature plaques^55^. Moreover, the actin cytoskeleton and its dynamics play pivotal roles in modulating synaptic function by organizing the PSD, anchoring postsynaptic receptors, facilitating the trafficking of synaptic cargoes, and localizing the translation machinery in the synapses^56,57^. Therefore, impairments in signaling pathways that regulate synaptic markers such as PSD-95 and synaptophysin, and actin dynamics, such as profilin-1, could lead to the synaptic and cognitive deficits observed early in AD.

Dendritic spines are specialized anatomical structures in neuronal cells that serve as the postsynaptic element/component for the vast majority of CNS synapses. Structural changes at dendritic spines underlie learning and memory processes in the brain, and changes in spine structure and function might lead to cognitive impairments. Spines can be classified morphologically into four types: stubby, mushroom, thin and filopodia-like, which corresponds to their maturation and function as immature, stable and mature, transient or lacking synapses, respectively^58,59^. The structure and function of dendritic spines are dynamically regulated by cellular pathways acting on the actin cytoskeleton. In the data reported here, we observed that an increase in Aβ42 both intra- and extracellularly was coupled with a reduction in important synaptic proteins and changes in the density and morphology of dendritic spines that may constitute the primary cause for the synaptic inhibition and memory impairments.

Synaptic deficits and synapse loss occur early in AD and MCI, before the onset of plaques, being some of the first signs of the neurodegenerative process^60,61^. As Aβ aggregates it can adopt different shapes such as fibrils and non-fibrillar aggregates^62,63^, and there is a consensus that Aβ alone is not the main mediator responsible for AD. However, its precise pathogenic roles, subcellular location and state are still being discussed. Here we have provided evidence that low intraneuronal accumulation of Aβ peptides provoked by hippocampal infusion of UBI-Aβ42, as well as combined intraneuronal and extracellular accumulation of Aβ provoked by BRI-Aβ42, can disrupt cognitive behavior, synaptic plasticity and spine morphology.

Some insights on the relationship of different amyloid structures have already been discussed^64–66^. Further studies are required to determine the roles of these AAV vectors in promoting specific Aβ isoforms. It is of great importance to the advancement of AD research the acknowledgment of how the available tools work, so they can be appropriately employed in order to provide relevant results.

## Methods

### Infusion of vectors

Stereotaxic injection of AAV-EGFP, AAV-BRI-Aβ42 and AAV-UBI-Aβ42 into the hippocampus was performed according to previously described surgical protocols^67,68^. Viral preps were generated as described previously^69^. Briefly, AAV vectors expressing the Aβ peptides under the control of the cytomegalovirus enhancer/chicken beta actin (CBA) promoter, a WPRE, and the bovine growth hormone polyA were generated by plasmid transfection with helper plasmids in HEK293T cells. 48 hours after transfection cells were harvested and lysed in the presence of 0.5% Sodium Deoxycholate and 50U/ml Benzonase (Sigma) by freeze thawing, and the virus isolated using a discontinuous Iodixanol gradient, and affinity purified on a HiTrap HQ column (Amersham). The genomic titer of each virus was determined by quantitative PCR.

4–5-month-old male C57/BL6 mice (purchased from the Jackson Laboratory, Maine, USA) were anesthetized and placed in stereotaxic frame under continuous isoflurane anesthesia. Using a 10-μl Hamilton syringe and 30-gauge needle, mice received 2-μl injections of AAV-EGFP, AAV-BRI-Aβ42 and AAV-UBI-Aβ42 (1 × 1010 genome particles/ul) in the right and left hemispheres at the following stereotaxic coordinates: anterior-posterior (AP) −2.06 mm; dorsoventral (DV) −1.95 mm; mediolateral (ML) ± 1.75 mm. Animals were allowed to recover on a heating pad before being placed back in their home cages. All animal procedures are in accordance with National Institutes of Health and University of California guidelines and were approved by the Use Committee at the University of California, Irvine.

### Cell culture

Primary hippocampal neurons were collected from postnatal day 0 C57BL/6 J mice. Cells were grown and fed twice a week with Neurobasal media with antibiotics and supplemented with GlutaMAX and B-27 (ThermoFisher Scientific). For experiments presented in Fig. 1a, cells were incubated for 24 h with AAV-EGFP, AAV-BRI-Aβ42 ([1] 4.7 × 1010 genome particles/ml, [2] 9.3 × 1010 genome particles/ml, [3] 2.8 × 1011 genome particles/ml) and AAV-UBI-Aβ42 ([1] 1.5 × 1010 genome particles/ml, [2] 7.7 × 1010 genome particles/ml, [3] 4.6 × 1011 genome particles/ml) and the media was collected and analyzed for Aβ42 levels with a sandwich ELISA system as described previously^70^. For experiments presented in Fig. 1b,c, cells were transduced with AAV-EGFP 7.6 × 1010 genome particles/ml, AAV-BRI-Aβ42 2.2 × 1011 genome particles/ml, and AAV-UBI-Aβ 1.6 × 1012 genome particles/ml. 72 h later, media was collected for analysis and cells were washed with ice-cold PBS. Then, M-PER complemented with proteases and phosphatases inhibitors (ThermoFisher Scientific) was added and cells were scrapped. Media and lysates were centrifuged at 12,000xg for 10 min at 4 °C. Protein concentration in the lysates was determined using a commercial Bradford assay (Biorad). Media and cell lysates were analyzed for high molecular Aβ oligomers using an ELISA kit (Wako, cat# 298–80101) following the manufacturer’s protocol.

### Aβ derived diffusible ligand (ADDL) preparation

ADDLs were prepared according to previous publications^71^. Briefly, Aβ1–42 was dissolved in hexafluoro-2-propanol (HFIP) and aliquoted to microcentifuge tubes. HFIP was removed by evaporation under vacuum and an aliquot of Aβ42 was dissolved in anhydrous dimethyl sulfoxide (DMSO), which was then added to ice-cold F12 medium without phenol red. This solution was incubated at 4 °C for 24 h and then centrifuged at 14 000 g for 10 min. Centrifugation produced a small pellet and the supernatant is defined as the ADDL preparation, which comprises fibril-free solutions of oligomers as well as monomers. Cells were incubated with 10 mM ADDLs for 24 h.

### Morris Water Maze

Three months after infusion of vectors, behavioral analyses were performed. Mice were trained to swim to a circular clear Plexiglas platform submerged 1.5 cm beneath the water’s surface. Four trials were performed per day, for 60 seconds each with 5 minutes between trials. Mice were trained for as many days as needed for the group to reach the training criterion of 25 seconds. The probe test was assessed 24 hours after the last trial, with the platform removed. Performance was monitored with the EthoVision XT video-tracking system (Noldus Information Technology, Leesburg, VA, USA).

### Contextual Fear Conditioning

During training, mice were placed in the fear conditioning chamber and allowed to explore for 2 minutes before receiving three electric foot shocks (duration: 1 s, intensity: 0.2 mA, intershock interval: 2 minutes). Animals were returned to the home cage 30 seconds after the last foot shock. Twenty-four hours later, behavior in the conditioning chamber was video recorded for 5 minutes and subsequently analyzed for freezing behavior.

### Fluorescence analysis of single-synapse long-term potentiation (FASS-LTP)

Activity-dependent responses in hippocampal synaptosomes were analyzed by FASS-LTP, as previously described^26,27^. FASS-LTP consists of chemical LTP (cLTP) stimulation directly in crude P2 synaptosomal fractions, immunofluorescence labeling for surface GluA1 and neurexin-1β, and flow cytometry analysis. Briefly, fresh crude synaptosome P2 fractions were obtained from the hippocampi of mice injected with AAVs and stimulated with glycine (500 μM) and KCl (50 mM). For surface immunolabeling, primary antibodies were rabbit anti-GluA1 (Cell Signaling #13185; 1:400) and mouse anti-Nrx1β (UC Davis/NIH NeuroMab Facility, 75–216; 2.5 μg/ml). Secondary antibodies were anti-rabbit-Alexa-405, anti-rabbit-Alexa-488 and anti-mouse-Alexa-647 antibodies (Life Sciences), at 2.5 μl/ml. Samples were protected from light, maintained at 4 °C and immediately run on a flow cytometer (Novocyte, ACEA Biosciences, Inc); 20,000 events were collected and analyzed for each sample with an event rate of approximately 500/sec. Analysis was performed using the FlowJo v10.3 software (LLC).

### Golgi staining

Following transcardial perfusion with 0.1 M phosphate-buffered saline (PBS, pH 7.4), mice brains were removed and processed using a superGolgi Kit (Bioenno Tech LLC, Santa Ana, CA), as described previously^24,72^

### Dendritic and spine analysis

Stereological quantifications were performed using Neurolucida software from Microbrightfield Bioscience (MBF Bioscience, Williston, VT, USA) to determine the number of spines in the stratum radiatum (SR) and the molecular layer (ML) of the hippocampal CA1 region, respectively. Briefly, every second section was used through the entire antero-posterior extent of the hippocampus (between −1.46 mm anterior and −3.40 mm posterior to Bregma according to Franklin and Paxinos, Third Edition, 2007). The SR and ML in the CA1 region were defined using a 5x objective and spines were counted using a 100x/1.4 objective. The coefficient of error (CE) value for each animal ranged between 0.03 and 0.08. Dendritic spine length was traced using a 100x/1.4 objective and data were analysed via Neurolucida Explorer software. For dendritic morphological analysis, 5 neurons per animal (n = 6) in the CA1 hippocampal area were traced using Neurolucida software. Dendritic width was measured using Image J software in electronic microscopic images (10 images per animal for a total of 6 mice per group).

### Immunoblotting

Equal amounts of protein (30 μg) were separated on 10% Bis-Tris gel (Invitrogen, Carlsbad, CA), and transferred to nitrocellulose membranes that were blocked in a 5% (w/v) suspension of Bovine Serum Albumin (BSA; Gemini Bio-Products, West Sacramento, CA, USA) in 0.2% Tween 20 Tris-buffered saline (TBS-T, pH 7.5) for 1 h. Next, membranes were incubated overnight at 4 °C with the following primary antibodies: synaptophysin 1:1000 (Abcam, Cambridge, UK), PSD-95 1:1000 (Abcam, Cambridge, UK), profilin-1 1:1000 (Abcam, Cambridge, UK) and GAPDH 1:1000 (Santa Cruz Biotechnology, CA, USA). Membranes were then washed in Tween-TBS for 20 min and incubated with specific secondary antibodies at a dilution of 1:10,000 (Pierce Biotechnology) for 60 min. Immunocomplexes were visualized using Super Signal (ThermoFisher Scientific, Rockford, IL, USA) and band density measurements were made using ImageJ imaging software version 1.36b (NIH).

### Immunohistochemistry

For immunohistochemistry, sections (40μm thick) were pretreated with 3% H2O2/3% methanol in Tris-buffered saline (TBS) for 30 min, followed by a TBS wash. Sections were then incubated in TBS with 0.1% Triton X-100 (TBST) for 15 min, followed by TBST with 2% BSA (Sigma-Aldrich) for 30 min. Sections were incubated with anti-6E10 (1:1000; Biolegend, San Diego, CA, USA) in TBS + 5% normal horse serum overnight at 40 C. Sections were then incubated with the appropriate secondary biotinylated antibody (1:500) in TBS containing 2% BSA plus 5% normal serum for 1 hour at room temperature, followed by Vector ABC Kit and DAB reagents (Vector Laboratories, Burlingame, CA, USA) to visualize staining.

### Electrochemiluminescence-linked immunoassay

Quantitative biochemical analyses of human Aβ and inflammatory cytokines in mouse tissue were performed using a commercially available electrochemiluminescence-linked immunoassay from Meso Scale Discovery (MSD, Gaithersburg, MD, USA). The V-PLEX Aβ Peptide Panel 1 (6E10) was used and plates were analyzed on the MS2400 imager (MSD). Assays were performed according to the manufacturer’s instructions, and all standards and samples were measured in duplicate.

### Enzyme-linked immunosorbent assay for Aβ42

Aβ1–42 was measured in the primary neuronal hippocampus cell culture medium using a sensitive sandwich enzyme-linked immunosorbent assay system as previously described^70^.

### Statistical analysis

All data between two groups were analyzed by Student’s *t*-test comparisons, and one- or two-way analysis of variance (ANOVA), followed by Bonferroni’s test for comparisons among more than 2 groups. Mann-Whitney U Test was used for the FASS-LTP data. Graphpad Prism software (Graphpad Prism Inc., San Diego, CA, USA) was used, and the significance was set at 95% of confidence. Values are presented as mean ± SEM.

## Data Availability

The datasets used and/or analyzed during the current study are available from the corresponding author on reasonable request.
